# Knock-In Rat Lines with Cre Recombinase at the Dopamine D1 and Adenosine 2a Receptor Loci

**DOI:** 10.1523/ENEURO.0163-19.2019

**Published:** 2019-09-13

**Authors:** Jeffrey R. Pettibone, Jai Y. Yu, Rifka C. Derman, Thomas W. Faust, Elizabeth D. Hughes, Wanda E. Filipiak, Thomas L. Saunders, Carrie R. Ferrario, Joshua D. Berke

**Affiliations:** 1 Department of Neurology, University of California, San Francisco 94143, CA; 2 Department of Physiology, University of California, San Francisco 94143, CA; 3 Department of Psychiatry, University of California, San Francisco 94143, CA; 4 Kavli Institute for Fundamental Neuroscience, University of California, San Francisco 94143, CA; 5 Weill Institute for Neurosciences, University of California, San Francisco 94143, CA; 6 Neuroscience Graduate Program, University of Michigan, Ann Arbor 48109, MI; 7 Transgenic Animal Model Core, University of Michigan, Ann Arbor 48109, MI; 8 Department of Internal Medicine, University of Michigan, Ann Arbor 48109, MI; 9 Department of Pharmacology, University of Michigan, Ann Arbor 48109, MI

**Keywords:** knockin rat, Cre rat line, dopamine, adenosine, striatum, direct pathway, indirect pathway

## Abstract

Genetically modified mice have become standard tools in neuroscience research. Our understanding of the basal ganglia in particular has been greatly assisted by BAC mutants with selective transgene expression in striatal neurons forming the direct or indirect pathways. However, for more sophisticated behavioral tasks and larger intracranial implants, rat models are preferred. Furthermore, BAC lines can show variable expression patterns depending upon genomic insertion site. We therefore used CRISPR/Cas9 to generate two novel knock-in rat lines specifically encoding Cre recombinase immediately after the dopamine D1 receptor (*Drd1a*) or adenosine 2a receptor (*Adora2a*) loci. Here, we validate these lines using *in situ* hybridization and viral vector mediated transfection to demonstrate selective, functional Cre expression in the striatal direct and indirect pathways, respectively. We used whole-genome sequencing to confirm the lack of off-target effects and established that both rat lines have normal locomotor activity and learning in simple instrumental and Pavlovian tasks. We expect these new D1-Cre and A2a-Cre rat lines will be widely used to study both normal brain functions and neurological and psychiatric pathophysiology.

## Significance Statement

This work presents the generation and validation of two novel knock-in rat lines. We demonstrate that the Cre transgene was correctly inserted at the intended genomic locations only and that the rats show normal behavior in a range of simple tests. We validate that Cre is expressed with high specificity and consistency and produces functional Cre-dependent protein expression *in vivo*. Among other applications, these lines will be valuable tools for selective investigations of the striatal direct and indirect pathways.

## Introduction

Dopamine and adenosine are important chemical messengers in the brain, vasculature, and elsewhere in the body. Within the brain, one key site of action is the striatum (including nucleus accumbens), a critical component of basal ganglia circuitry involved in movement, motivation, and reinforcement-driven learning (Denny‐Brown and Yanagisawa, 1976; Marsden, 1982; Gerfen and Surmeier, 2011; Berke, 2018). Most (90–95%) striatal neurons are GABAergic medium spiny neurons (MSNs) with two distinct subclasses (Gerfen and Surmeier, 2011). “Direct pathway” neurons (dMSNs) express dopamine-D1 receptors and project primarily to the substantia nigra pars reticulata/globus pallidus pars interna (SNr/GPi), whereas “indirect pathway” neurons (iMSNs) express both dopamine-D2 receptors and adenosine-A2a receptors, and project primarily to the globus pallidus pars externa (GPe). Although our understanding of their distinct functions is incomplete, dMSNs and iMSNs have complementary roles promoting and discouraging motivated behaviors, respectively (Collins and Frank, 2014).

The investigation of dMSNs and iMSNs has been transformed by transgenic mice. Random genomic insertion of BACs (bacterial artificial chromosomes) encoding dopamine receptor promoters driving fluorescent protein expression confirmed the near-total segregation of striatal D1 and D2 receptors (Shuen et al., 2008; Matamales et al., 2009) and enabled identification of dMSNs/iMSNs in brain slices (Day et al., 2006). BAC lines in which dopamine receptor promoters drive Cre recombinase expression (D1-Cre, D2-Cre, etc.) have allowed *in vivo* identification and manipulation of neuronal subpopulations in striatum (Kravitz et al., 2010, 2012; Cui et al., 2013; Barbera et al., 2016) and cortex (Kim et al., 2017). iMSNs targeting is further improved using an A2a promoter, rather than D2, because A2a receptors are selectively expressed on iMSNs while D2 receptors are also expressed on other striatal cells and synapses (Alcantara et al., 2003).

However, for many experiments, rats are more suitable than mice. Their larger size means they can bear complex intracranial implants without loss of mobility. Furthermore, rats can learn more sophisticated behavioral tasks, including those investigating reinforcement learning (Hamid et al., 2016) and behavioral inhibition (Schmidt et al., 2013). The advent of CRISPR/Cas9 methods has facilitated the generation of knock-in rat lines (Mali et al., 2013; Jung et al., 2016), and knock-ins are more likely to have faithful expression patterns compared to BACs for which (for example) different D1-Cre lines show markedly different expression (Heintz, 2004).

Here, we describe the generation of transgenic D1-Cre and A2a-Cre rat lines using CRISPR/Cas9. We then demonstrate the specificity of *iCre* mRNA expression in the intended cells, in both dorsal striatum (DS) and nucleus accumbens. Next, we confirm Cre-dependent expression to demonstrate that Cre is functional and appropriately confined to the direct or indirect pathways. Finally, we demonstrate normal locomotor activity, learning and motivation in simple behavioral tasks.

## Materials and Methods

All animal procedures were approved by the relevant Institutional Animal Care and Use Committees.

### Genetic engineering

CRISPR/Cas9 (Mali et al., 2013) was used to generate genetically-modified rat strains. Two single guide RNA (sgRNA) targets and protospacer adjacent motifs (PAMs) were identified downstream of the rat *Adora2a* termination codon (Hsu et al., 2013). sgRNA targets were cloned into plasmid pX330 (Addgene #42230, a gift of Feng Zhang) as described (Ran et al., 2013). Guide targets were C30G1: CTAAGGGAAGAGAAACCCAA PAM: TGG, and C30G2: GGCTGGACCAATCTCACTAA PAM: GGG. Purified pX330 plasmids were co-electroporated into rat embryonic fibroblasts with a PGKpuro plasmid (McBurney et al., 1994). Genomic DNA was prepared after transient selection with puromycin (2 µg/ml). A 324-bp DNA fragment spanning the expected Cas9 cut sites was PCR-amplified with forward primer GGGATGTGGAGCTTCCTACC and reverse primer GCAGCCCTGACCTAACACAG. DNA sequencing of the amplicons showed that C30G1-treated, but not C30G2-treated, cells contained overlapping chromatogram peaks, indicative of multiple templates that differ because of non-homologous end-joining repair of CRISR/Cas9-induced chromosome breaks resulting in the presence of small deletions/insertions (indels). sgRNA C30G1 was chosen for rat zygote microinjection. A DNA donor was synthesized (BioBasic, cloned in pUC57) to introduce the following elements between codon 410 and the termination codon of *Adora2a*: a glycine-serine-serine linker with porcine teschovirus-1 self-cleaving peptide 2A (P2A; Kim et al., 2011) followed by iCre recombinase (Shimshek et al., 2002) with hemagglutinin tag YPYDVPDYA (Kolodziej and Young, 1991) and a termination codon with the bovine growth hormone polyadenylation sequence (Goodwin and Rottman, 1992). To mediate homologous recombination a 5’ arm of homology (1804 bp of genomic DNA 5’ to codon 410) and a 3’ arm of homology (1424 bp of genomic DNA downstream of the termination codon) were used. The 20-bp sequence of C30G1 was omitted from the 3’ arm of homology to prevent CRISPR/Cas9 cleavage of the chromosome after insertion of the DNA donor.

A similar approach was used for *Drd1a*. Two sgRNA were identified downstream of the *Drd1a* termination codon, C31G1: TTCCTTAACAGCAAGCCCAA PAM: GGG and C31G2: CTGAGGCCACGAGTTCCCTT PAM: GGG. A 293-bp DNA fragment spanning expected Cas9 cut sites was PCR-amplified with forward primer TGGAATAGCTAAGCCACTGGA and reverse primer CTCCCAAACTGA TTTCAGAGC. Both sgRNAs were found to be active after transfection in rat fibroblasts by T7 endonuclease 1 (T7E1) assays (Sakurai et al., 2014). Briefly, DNA amplicons were melted and re-annealed, then subjected to T7EI digestion. The presence of indels produced by non-homologous endjoining repair of Cas9-induced double strand breaks resulted in the presence of lower molecular weight DNA fragments for both sgRNA targets, and C31G1 was chosen for zygote microinjection. A DNA donor was synthesized (BioBasic, cloned in pUC57) to introduce the following elements between *Drd1a* codon 446 and the termination codon: a glycine-serine-serine linker with P2A followed by *iCre* recombinase with V5 peptide tag GKPIPNPLLGLDST (Yang et al., 2013) and a termination codon with the bovine growth hormone polyadenylation sequence. To mediate homologous recombination a 5’ arm of homology (1805 bp of genomic DNA 5’ of codon 446) and a 3’ arm (1801 bp of genomic DNA downstream of the termination codon) were used. The 20-bp sequence of C31G1 was omitted from the 3’ arm of homology to prevent cleavage of the chromosome after insertion.

Rat zygote microinjection was conducted as described (Filipiak and Saunders, 2006). sgRNA molecules from a PCR-amplified template were obtained by *in vitro* transcription (MAXIscript T7 Transcription kit followed by MEGAclear Transcription Clean-Up kit, Thermo Fisher Scientific). The template was produced from overlapping long primers (IDTDNA) that included one gene-specific sgRNA target and T7 promoter sequence that were annealed to a long primer containing the sgRNA scaffold sequence (Lin et al., 2014). Cas9 mRNA was obtained from Sigma-Aldrich. Circular DNA donor plasmids were purified with an endotoxin-free kit (QIAGEN).

Knock-in rats were produced by microinjection of a solution containing 5 ng/μl Cas9 mRNA, 2.5 ng/μl sgRNA, and 10 ng/μl of circular donor plasmid. Before rat zygote microinjection, fertilized mouse eggs were microinjected with the nucleic acid mixtures to ensure that the plasmid DNA mixtures did not cause zygote death or block development to the blastocyst stage. Rat zygotes for microinjection were obtained by mating superovulated Long–Evans female rats with Long–Evans male rats from an in-house breeding colony. A total of 353 rat zygotes were microinjected with A2a-Cre reagents, 289 survived and were transferred to pseudopregnant SD female rats (Strain 400, Charles River), resulting in 60 rat pups; 401 rat zygotes were microinjected with D1-Cre reagents, 347 survived and were transferred, resulting in 95 pups. Genomic DNA was purified from tail tip biopsies (QIAGEN DNeasy kit) to screen potential founders for correct insertion of *iCre*.

### Colony management and genotyping

Lines were maintained by backcrossing with wild-type Long–Evans rats (Charles River or Harlan). Offspring were genotyped using real-time PCR (Transnetyx), using insertion-spanning primers (Table 2).

Genome sequencing was performed at the UCSF Institute for Human Genetics using blood samples (1 ml per rat) from 5th generation backcrossed rats. Libraries were prepared from fragmented DNA (Kapa Hyper Prep) and sequenced (Illumina NovaSeq 6000, S4 flow cell, paired-end mode, read lengths 150 bp). Sequencing reads were aligned to the rat genome (RGSC Rnor_6.0) using the Burrows-Wheeler Aligner (BWA-MEM). We used GATK HaplotypeCaller, Samtools, Bedtools, Pysam, and MATLAB for variant calling, subsequent analysis and visualization.

To determine the location of the inserted *iCre* cassette, we selected reads that did not align as a pair to the rat genome, which includes reads where only one mate or no mate of the pair aligned to the genome. These unaligned reads will include matches to the inserted *iCre* cassette sequence, which is not part of the reference genome. We searched for paired-end reads where one mate is aligned to the *iCre* cassette and then examined where in the genome the other mate is aligned.

To further verify the integrity of our lines we examined potential off-targets (D1-Cre: 197 sites and A2a-Cre: 557 sites) predicted by an *in silico* sgRNA off-target prediction algorithm (CRISPOR.net RSGC Rnor_6.0). CRISPR/Cas9-induced mutations in exons are of particular concern. Only two potential off-targets were predicted to be in exons in the D1-Cre line and none in the A2a-Cre line. After analyzing assembled genomic sequence data, we found the D1-Cre sequence contained a one base pair deletion at chr18:49935989 (Zfp608) and a single nucleotide variant (SNV) at ch1:258074844 (Cyp2c). On inspection, these changes were present in both the D1-Cre line and the A2a-Cre line, consistent with natural variations in the Long–Evans strain rather than off-target mutations from the D1-targeting sgRNA. Among the 195 predicted intronic off-targets for the D1-Cre strain located in introns, we observed 11 changes (eight contained SNVs and three contained indels). For the 557 predicted intronic off-target locations in the A2a-Cre strain, 48 locations showed changes (39 contained SNVs, six contained indels, and three contained both). Closer inspection of the indels revealed that 100% were present in both D1-Cre and A2a-Cre lines. This is again consistent with Long–Evans strain variation rather than off-target changes.

### *In situ* hybridization

Frozen brains (*n* = 6, one male and two females from each line) were stored at –80°C (overnight, two weeks), then sectioned on a cryostat at 20 μm and mounted on glass slides. Sections were fixed in 4% PFA at 4°C for 15 min and dehydrated through 50%, 75%, 100% and fresh 100% EtOH at room temperature (RT) for 5 min each. Slides were dried completely for 5 min. A hydrophobic barrier (Advanced Cell Diagnostics) was drawn around each section. Slides were rinsed twice in 1× PBS (∼1–3 min) and incubated with Protease IV reagent (Advanced Cell Diagnostics) for 30 min at RT. Fluorescent probes (RNAScope, Advanced Cell Diagnostics, *iCre* catalog 312281, *Drd1a* catalog 317031-C2, and *Adora2a* catalog 450471-C3) were added (2 h, 40°C) followed by manufacturer-specified washing and amplification. DAPI was added to the slides before coverslipping (Prolong Gold, Thermo Fisher Scientific).

We used MIPAR software (https://www.mipar.us) to segment cell boundaries and fluorescent puncta using separate processing pipelines. To define nuclear boundaries, the DAPI channel of each image was first histogram-equalized to compensate for uneven illumination (512 × 512 pixel tiles) and convolved with a pixel-wise adaptive low-pass Wiener filter (5 × 5 pixel neighborhood size) to reduce noise. The image was then contrast-adjusted (saturating the top and bottom 1% of intensities). Bright objects were segmented using an adaptive threshold (pixel intensity >110% of mean in the surrounding 30-pixel window). Image erosion followed by dilation further reduced noise (five-pixel connectivity threshold, 10 iterations). The Watershed algorithm was applied to improve object separation. Objects >5000 pixels (i.e., clustered nuclei) were identified and reprocessed to improve separation. Since mRNA fluorescent puncta can be located in the endoplasmic reticulum, we dilated the boundaries of each segmented nucleus by five pixels to include these regions.

To segment fluorescent puncta, each of the three probe channels were first preprocessed using a Top-hat filter (9- to 15-pixel radius), Wiener filter (15 × 15 pixel neighborhood size) followed by contrast adjustment (saturating top and bottom 1% of intensities). Bright regions were segmented using the extended-maxima transform (8-connected neighborhood, 5 H-maxima). A Watershed algorithm followed by erosion was used to improve object separation. Objects less than five pixels were rejected as noise. The location of each punctum is defined as the centroid of the segmented object.

For each fluorescent probe image channel, we counted the number of segmented puncta lying within a nuclear boundary. To determine the puncta threshold for specific versus non-specific probe hybridization, we estimated the “baseline” number of puncta expected per nucleus by chance from non-specific hybridization. We first calculated the puncta count per pixel for all puncta lying outside of cell nuclei and then multiplied this value by the number of pixels for each DAPI-labeled nucleus. This background puncta count was assumed to follow a Poisson distribution, and we defined our threshold for categorizing a cell as “positive” for a given mRNA probe as the 95th percentile of this distribution. Consistency was calculated as the percentage of *Drd1a+* (or *Adora2a+,* in the case of A2a-Cre) nuclei that are also positive for *iCre*. Specificity was calculated as the percentage of *iCre+* nuclei that are also *Drd1a+* (or *Adora2a+*). Off-target consistency and specificity were calculated the same way but substituting *Drd1a*+ (or *Adora2a*+) for each other in the above two equations.

### Virus injection

Rats (*n* = 2 females, one from each line) were microinjected with 0.5 μl of AAV5-CAG-Flex-TdTomato virus (UNC Vector Core) in DS at three locations along a dorsal-ventral trajectory (AP: +1.5, ML: +2.2, DV: –3.0, –4.0, –5.0 from brain surface), and killed four weeks after surgery.

### *In vivo* opto-tagging

Rats (*n* = 2, one male from each line) were injected with 1.0 μl of hSyn-Flex-ChrimsonR-TdTomato virus (UNC Vector Core) bilaterally in ventral striatum (AP: +1.75, ML: ±1.6, DV: –7.0 from brain surface) and implanted with two 64-channel drivable tetrode arrays, each with a fixed optical fiber extending centrally through the array to a depth of 6.5 mm. After three weeks of transfection, the tetrodes were lowered into the ventral striatum and recorded wideband (1–9000 Hz) at 30,000 samples/s using an Intan digital headstage. Recording ended with a brief laser stimulation protocol (1 mW, 638 nm, 1–10 ms/1 Hz). The rat was awake, unrestrained, and resting quietly throughout the recording.

Units were isolated offline using automated spike sorting software (MountainSort; Chung et al., 2017) followed by manual inspection. For a unit to be considered a successfully-identified Cre+ neuron it had to meet several criteria: (1) evoked spiking within 10 ms of laser onset, that reached the *p* < 0.001 significance level in the stimulus-associated latency test (Kvitsiani et al., 2013); (2) peak firing rate (*z*-scored) of >10 during both 5- and 10-ms laser pulses; (3) a Pearson correlation coefficient >0.9 between their average light-evoked wave form and their average session-wide wave form.

### Imaging

Images were taken with a Nikon spinning disk confocal microscope with a 40× objective (Plan Apo Lambda NA 0.95). For viral tracing, images (2048 × 2048 pixels at 16-bit depth) were stitched in FIJI.

### Behavior

Rats were maintained on a reverse light-dark schedule (12/12), testing was conducted during the dark phase, and rats were at least 70 d old at the start of the studies. Males and females were used for instrumental and Pavlovian studies. Males were used to evaluate cocaine-induced locomotor activity because of well-established sex differences in response to cocaine (Becker and Koob, 2016) and an insufficient available number of females to examine them separately.

For instrumental and Pavlovian procedures, procedures were conducted in operant chambers as described (Derman and Ferrario, 2018). Rats [D1-Cre–, *n* = 9 (three males, six females); D1-Cre*+*, *n* = 16 (seven males, nine females); A2a-Cre–, *n* = 8 (six males, two females); A2a-Cre*+*, *n* = 16 (10 males, six females)]. were food restricted to 85–90% of free-feeding body weight. For instrumental training, a food cup was flanked by two retractable levers. First, rats were given two sessions in which 20 food pellets (45 mg, Bioserv #F0021) were delivered into the food cup on a variable-interval schedule of 60 s (VI60). Next, rats underwent instrumental training in which responses on the “active” lever resulted in delivery of a single pellet (fixed ratio 1; FR1) and responses on the other ‘inactive’ lever had no consequences. Rats were trained to an acquisition criterion of 50 pellets within 40 min. The same rats then underwent Pavlovian conditioning using two auditory conditioned stimuli (CSs; tone and white noise, 2 min; four presentations of each CS per session, 5 min ITI, 12 sessions, 1 h/session). Fifteen seconds following CS+ onset, four pellets were delivered on a VI30 schedule. The CS– was presented an equal number of times, unpaired with pellets. Food cup entries were recorded in 10-s bins and entries during the first 10 s of CS presentations were used to evaluate conditioned anticipatory responding [i.e., before unconditioned stimulus (US) delivery].

Locomotor activity was assessed in a subset of the rats trained above (Cre–, *n* = 7; D1*-*Cre*+*, *n* = 7; A2a-Cre*+*, *n* = 7) using procedures similar to (Vollbrecht et al., 2016). Rats were allowed to feed freely for at least 5 d before locomotor testing. Testing was conducted in rectangular plastic chambers (25.4 × 48.26 × 20.32 cm) outfitted with photocell arrays around the base perimeter. Beam breaks were measured using CrossBreak software (Synaptech; University of Michigan). Rats were habituated to the testing chambers (30 min) and given two injections of saline (1 ml/kg, i.p.) separated by 45 min. Next, the acute locomotor response to cocaine was assessed. After a 30-min habituation, rats were given a saline injection followed 45 min later by cocaine (15 mg/kg, i.p.) and remained in the chambers for an additional 60 min. Locomotor activity was recorded in 5-min bins throughout and reported as crossovers (beam break at one end of the cage followed by beam break at the opposite end of the cage).

## Results

### Molecular design

**Figure 1.**
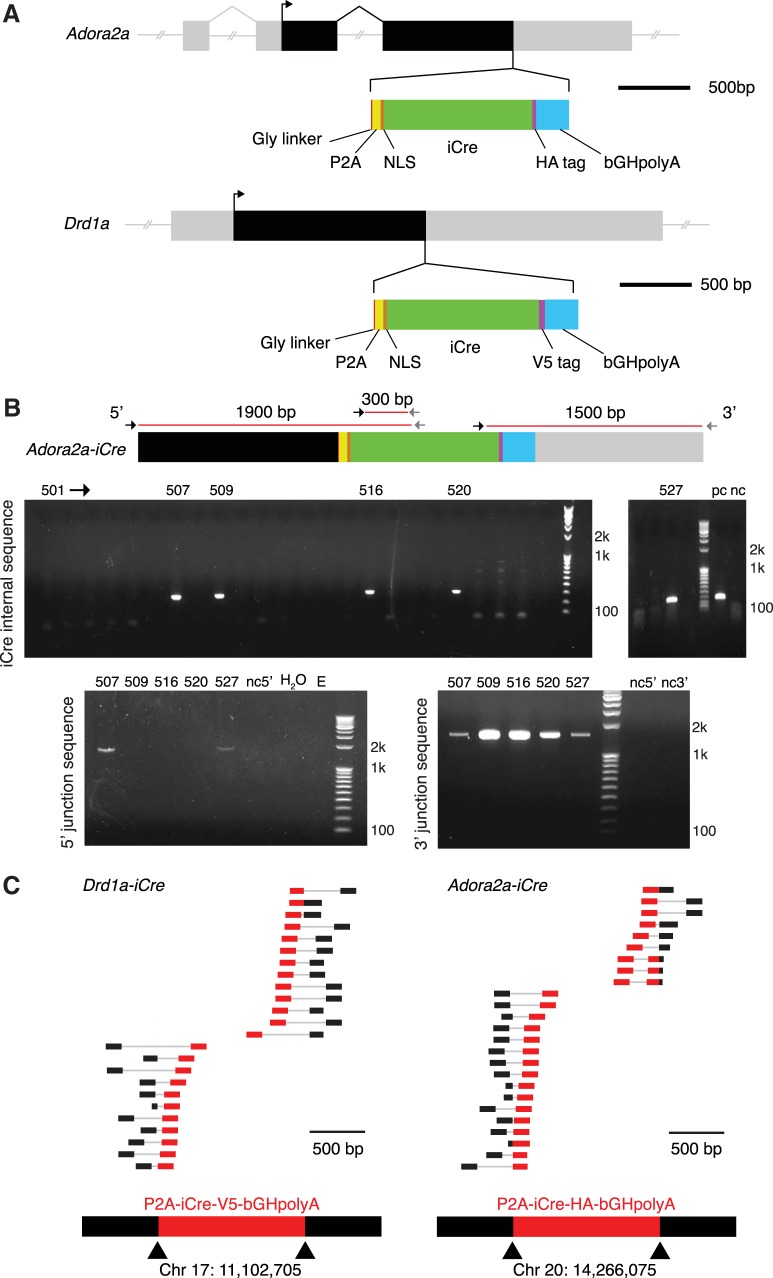
Details of insertion design and founder line screening. ***A***, Schematic of insertion cassettes into *Adora2a* (above) and *Drd1a* (below) genes. NLS, nuclear localization sequence; HA, influenza hemagglutinin protein tag YPYDVPDYA; V5, peptide tag GKPIPNPLLGLDST; bGH, bovine growth hormone polyadenylation sequence. ***B***, PCR primer loci (above) and corresponding gels (below) demonstrating G0 screening of the A2a-Cre line. The top row of gels indicate that rats 507, 509, 516, 520, and 527 are transgenic for *iCre*. The bottom gels show that rats 520 and 527 have *iCre* inserted correctly at both the 3’ and 5’ junctions. See Table 1 for full primer sequences for screening both lines. pc, single copy detection; nc, unrelated rat tail DNA; H2O, water control; E, empty. ***C***, Reads from whole genome sequencing aligned to a wild-type rat genome demonstrate that, for each transgenic line, the *iCre* cassette is inserted only once in the genome and at the target loci. Each row corresponds to one paired-end read, where one mate of the pair is aligned to the inserted cassette (red) and the other mate in the genome (black). Sequence reads with at least 100-bp match in the inserted cassette are shown. All such pairs map to only one location in the genome.

The D1-Cre and A2a-Cre rat lines were designed so that the native *Drd1a* or *Adora2a* promoter drives expression of both the native receptor and the codon-improved *Cre* recombinase (*iCre*) sequence in a single transcription event (Fig. 1*A*). The use of iCre over Cre has been shown to enhance recombinase expression and limit epigenetic silencing in mammalian cells (Shimshek et al., 2002). For each line, a unique single strand guide RNA (sgRNA) was generated to induce double strand breaks at the terminus of the receptor coding sequence and microinjected into Long–Evans rat zygotes along with Cas9 and a circular plasmid containing the donor gene cassette. After correct recombination of the donor cassette, the 3’ end of target receptor sequence will be joined in frame with the “self-cleaving” peptide P2A (to separate the Cre protein after translation), followed by Cre with a nuclear localizing signal affixed at the amino terminus, and a peptide tag (HA for Adora2a, V5 for D1) to facilitate antibody-based detection.

### Founder screening, germline transmission, and full genome sequencing

DNA samples from G0 potential founders were screened with primers to detect *iCre* in the genome (for primer sequences, see Materials and Methods). From this screen 21/96 potential D1-Cre, and 9/60 potential A2a-Cre founders were positive for *iCre*. Positive rats were then screened with additional primers across the junctions between native and introduced DNA stretches, to discriminate between correct and random genomic integration events (Fig. 1*B*). This yielded 7/21 correct D1-Cre insertions and 7/9 correct A2a-Cre insertions. The *iCre* insert was then completely sequenced in these rats (14 total) to confirm complete integration.

These G0 founders were mated with wild-type Long–Evans rats, and the G1 pups genotyped for *iCre* specific insertion as above to verify germline transmission. Colonies from one successful founder for each line were established and maintained by back-crossing to wild-type Long–Evans rats from commercial vendors (see Materials and Methods); all experimental results shown are from rats back-crossed for at least three generations.

After five generations of back-crossing, we took one female rat each from the D1-Cre and A2a-Cre lines and sequenced their entire genomes to confirm that *iCre* was present in the intended location and nowhere else (Fig. 1*C*). Average sequencing depths for D1-Cre and A2a-Cre lines were 80× (1,503,983,138 reads) and 71× (1,358,732,834 reads), respectively. To determine the location of the inserted gene cassette, we identified paired sequence reads for which one mate of the pair aligned to the rat genome (Rnor_6.0) and the other mate aligned to the inserted gene cassette. All such reads were aligned to the genome in the expected location in each line (24/24 D1-Cre, 25/25 A2a-Cre), indicating correct, single copy insertion (Fig. 1*C*).

Partial sequence matches between the sgRNA and genomic locations away from the intended target may induce “off-target” cleavage events. Any off-target changes are likely to be progressively diluted over successive generations of back-crossing. We nonetheless performed an extensive screen and found no evidence for off-target events (see Materials and Methods).

### Consistent and specific Cre expression in *Drda1*-expressing or *Adora2a*-expressing cells

**Figure 2.**
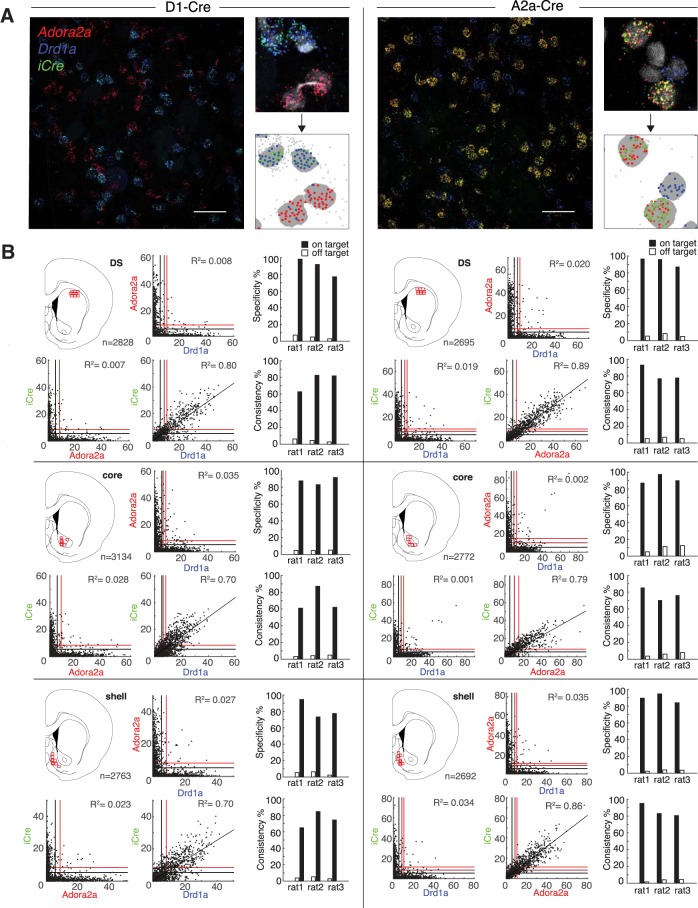
Confirmation and quantification of *iCre* production in D1+ and A2a+ MSNs. ***A***, left of each column, Example 40× images of FISH labeling used for quantification, taken from DS (scale bars = 50 μm). Right of each column: closeup images (top) aligned with their corresponding automated software output (bottom). Gray regions indicate DAPI boundaries and colored dots indicate puncta within DAPI boundaries, using the same color scheme as the raw images. Gray dots indicate the locations of puncta detected outside of DAPI boundaries. ***B***, Scatterplots of raw puncta counts for each cell show selective *iCre* mRNA co-localization with the target receptor mRNA. Black, dark red, and red lines indicate the 50th (i.e., median), 95th, and 99.9th confidence limits, respectively. Subpanels are grouped into rows by region and into columns by genotype. Atlas images depict the locations of confocal images used for mRNA quantification. Barplots show specificity and consistency of on-target and off-target expression, in each rat (*n* = 3 rats per line).

The knock-in design ought to produce *iCre* mRNA expression that is highly faithful to the natural distribution of *Drd1a* (or *Adora2a*) mRNA. To assess this, we used triple fluorescent *in situ* hybridization, together with DAPI labeling of cell nuclei. Probe sets targeting *iCre*, *Adora2a* receptor and *Drd1a* receptor mRNA with distinct color labels were multiplexed and visualized simultaneously (Fig. 2*A*). mRNA expression was quantified in three distinct striatal subregions, the DS, the nucleus accumbens core, and the nucleus accumbens medial shell. Automated software was used to define cell boundaries and count fluorescent puncta per cell, for each probe (Fig. 2*A*).

Relationships between puncta counts for *Drd1a*, *Adora2a*, and *iCre* are shown in Figure 2*B*, left column, D1-Cre, and right column, A2a-Cre. As expected, in D1-Cre rats expression of *Drd1a* and *iCre* mRNA was closely correlated in all striatal subregions examined (DS: *R* ^2^ = 0.80; core: *R* ^2^ = 0.70; shell: *R* ^2^ = 0.70; Fig. 2*B*), and there was no correlation between *Adora2a* and *iCre* mRNA (DS: *R* ^2^ = 0.008; core: *R* ^2^ = 0.035; shell: *R* ^2^ = 0.027). Conversely, in A2a-Cre rats expression of *Adora2a* and *iCre* mRNA was closely correlated (DS: *R* ^2^ = 0.89; core: *R* ^2^ = 0.79; shell: *R* ^2^ = 0.86), and there was no correlation between *Drd1a* and *iCre* mRNA (DS: *R* ^2^ = 0.019; core: *R* ^2^ = 0.001; shell: *R* ^2^ = 0.034). Consistent with earlier *in situ* hybridization studies (Le Moine and Bloch, 1995; Berke et al., 1998), we found near-complete segregation of dMSN and iMSN markers in all regions examined, with virtually no overlap between *Drd1a* and *Adora2a* expression (Fig. 2*A*,*B*).

To further assess the specificity and consistency of *iCre* mRNA expression we defined thresholds for considering neurons as positive for a given probe. Given the wide distributions of puncta counts, the choice of threshold is non-trivial; it forces a trade-off between Type I and Type II errors. Therefore, rather than picking an arbitrary threshold, for each probe we chose the 95% upper confidence limit, assuming a Poisson background distribution of puncta (see Materials and Methods). Using these thresholds (marked by red lines on the Fig. 2*B*, scatterplots) we estimated A2a-Cre specificity (% of *iCre+* that are also *Adora2a+*) to be 93.5% (DS), 91.8% (core), and 89.2% (shell), and consistency (% of *Adora2a+* that are also *iCre+*) to be 82.8% (DS), 77.4% (core), and 86.2% (shell). In the D1-Cre line, we estimated specificity (% of *iCre+* that are also *Drd1a+*) to be 89.1% (DS), 87.4% (core), and 81.8% (shell), and consistency (% of *Drd1a+* that are also *iCre+*) to be 77.5% (DS), 70.1% (core), and 74.6% (shell). If we use even higher thresholds for *Drd1a* and *Adora2a* (e.g., >30 puncta/cell), we can be essentially certain of cell identity and assessed this way consistency was close to 100% for both lines (Fig. 2*B*).

### Cre-dependent protein expression

**Figure 3.**
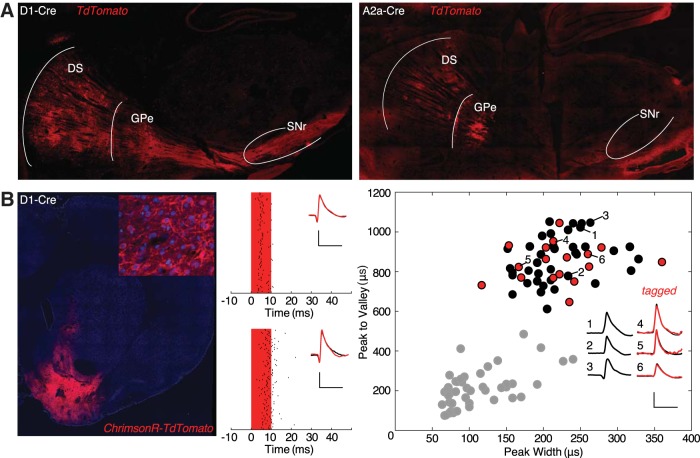
Cre-dependent expression confirms pathway segregation and functional expression ***A***, Functional Cre expression is confined to appropriate BG pathways. Left, CAG-Flex-tdTomato injected into DS of the D1-Cre line expresses in terminals in SNr/GPi. Right, CAG-Flex-tdTomato injected into DS of the A2a-Cre line expresses in terminals in GPe but not SNr/GPi. ***B***, Optogenetic identification of Cre+ cells. Left, hSyn-FLEX-ChrimsonR-TdTomato expression pattern into ventral striatum of a D1-Cre animal. Inset at top right, Closer-up view of transfected neurons. Middle, Examples of a light-responsive neuron in a D1-Cre rat (top; identified dMSN) and an A2a-Cre rat (bottom; identified iMSN). Red bar indicates duration of light pulse, small black bars indicate spike times surrounding each stimulation (rows). Inset, Average session-wide spike wave form (black) with average light-evoked wave form overlaid in red. Scale bars = 0.1 mV, 1 ms. Right, Wave form feature plot demonstrates that light-responsive dMSNs (red) are intermingled within the large cluster of presumed MSNs (black). Other, unclassified cells (mostly GABAergic interneurons) are shown in gray. Inset includes average spike waveforms from three examples each of light-responsive and non-responsive cells within the MSNs cluster. Scale bars = 0.1 mV, 1 ms.

We next examined whether *iCre* mRNA expression results in functional Cre protein confined to the appropriate basal ganglia pathway. To this end, we injected DS with a virus for Cre-dependent expression of a fluorescent protein (AAV-CAG-FLEX-tdTomato) and examined the expression pattern four weeks later. Consistent with pathway-specific expression of functional Cre protein, injection into the D1-Cre line resulted in clear expression in the striato-nigral pathway, while injection into the A2a-Cre line produced labeling in both DS and GPe, but no expression in the SNr (Fig. 3*A*).

One important use of Cre lines is to enable positive identification of recorded neuron subtypes in awake behaving animals (Kravitz et al., 2010), via Cre-dependent opsin expression and monitoring neuronal responses to light pulses. We found that both the D1-Cre and A2a-Cre rat lines can be used for this purpose. In rats from each line we injected a virus (AAV-hSyn-FLEX-ChrimsonR-Tdtomato) into the accumbens core for Cre-dependent expression of the red-shifted opsin Chrimson (Klapoetke et al., 2014; Fig. 3*B*, left) followed by a custom optrode (Mohebi et al., 2019). After allowing three weeks for opsin expression, we readily observed light-responsive single units (Fig. 3*B*, middle). In a representative example session from a D1-Cre rat 17 neurons were identified as dMSNs, as they showed both a reliable response to red light stimulation and the wave form properties typical of MSNs (Fig. 3*B*, right; Berke et al., 2004; Gage et al., 2010). As these cells were intermingled within the larger MSNs cluster, it would not have been possible to identify them without this optogenetic tagging procedure.

### Normal acquisition and performance of instrumental and Pavlovian discrimination and cocaine-induced locomotor activity

Given that behavioral comparisons are likely to be made across these two independent transgenic lines, and between Cre+ rats and Cre– controls, we assessed acquisition and expression of instrumental responding for food and Pavlovian conditioned approach, and cocaine-induced locomotor activity in these lines.

**Figure 4.**
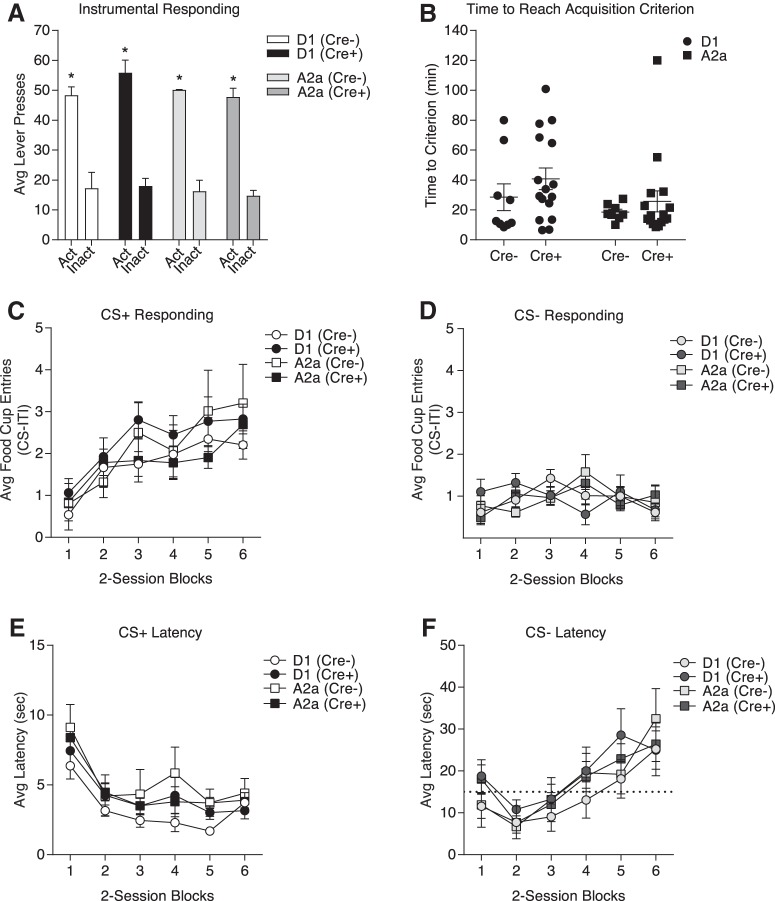
Instrumental and Pavlovian discrimination are similar between transgenic lines and Cre– littermate controls. ***A***, The average total number of responses on the active and inactive lever did not differ between groups and all groups preferentially responded on the active lever; *p < 0.05 active versus inactive responses. ***B***, The total time to reach the acquisition criterion does not differ between groups. ***C***, The average rate of food cup entries during the first 10 s of CS+ presentations increases across two-session training blocks and is similar between groups. ***D***, The average rate of food cup entries during the first 10 s of CS– presentations is low, does not change across training blocks and is similar between groups. ***E***, The average latency to approach the food cup following CS+ onset gets faster across training and is similar between groups. ***F***, The average latency to approach the food cup following CS– becomes slower across training and is similar between groups. Note the scale difference between panels ***E***, ***F***; the dotted line in panel ***F*** indicates 15 s on the *y*-axis to facilitate comparison. All data represented as mean ± SEM.

In the instrumental discrimination task presses on an active lever were reinforced with food pellet delivery (fixed ratio of 1; FR1), whereas presses on an inactive lever were never reinforced. Rats were trained to an acquisition criterion of earning 50 pellets within <40 min. Figure 4*A* shows the average number of active and inactive lever responses, and Figure 4*B* depicts the average time to reach the acquisition criterion in each group. As expected active lever responding was greater than inactive lever responding, and this did not differ between groups (two-way repeated-measures ANOVA, main effect of lever: *F*_(1,90)_ = 193.2, *p* < 0.0001; n.s. main effect of group: *F*_(3,90)_ = 1.379, *p* = 0.2545; n.s. group × lever interaction: *F*_(3,90)_ = 0.408, *p* = 0.747). The time to reach acquisition criterion did not differ between groups (two-way repeated-measures ANOVA, n.s. main effect of lineage: *F*_(1,45)_ = 2.593, *p* = 0.1143; n.s. main effect of genotype: *F*_(1,45)_ = 1.578, *p* = 0.2155; n.s. lineage × genotype interaction: *F*_(1,45)_ = 0.1086, *p* = 0.7433).

Following instrumental training, the acquisition and expression of Pavlovian conditioned approach were assessed in the same rats. During each session, one auditory cue was paired with food pellet delivery (CS: CS+), whereas a second auditory cue was never paired with food (CS–). Rats received 12 training sessions (60 min) in which each CS (tone or white noise, counterbalanced for CS+/CS– assignment) was randomly presented four times per session. Acquisition of Pavlovian conditioned food cup approach was similar across transgenic lines and between Cre– and Cre*+* groups. Specifically, Figure 4*C*,*D* shows the average number of food cup entries during the first 10 s of CS+ and CS– in two-session blocks, respectively. Anticipatory food cup entries during CS+ presentations increased across training blocks and did not differ between groups (two-way repeated-measures ANOVA, main effect of training block: *F*_(5,225)_ = 13.45, *p* < 0.0001; n.s. main effect of group: *F*_(3,45)_ = 0.505, *p* = 0.6807; n.s. group × training block interaction: *F*_(15,225)_ = 0.6086, *p* = 0.866; Fig. 4*C*). In contrast, food cup entries during the first 10 s of CS– presentations did not increase across sessions and was similar across groups (two-way repeated-measures ANOVA, n.s. main effect of training block: *F*_(5,225)_ = 1.602, *p* = 0.1606; n.s. main effect of group: *F*_(3,45)_ = 0.01628, *p* = 0.9971; n.s. group × training block interaction: *F*_(15,225)_ = 1.476, *p* = 0.1155; Fig. 4*D*). Thus, acquisition and maintenance of discriminatory conditioned approach were similar across transgenic lines, and between Cre– and Cre*+* groups.

To provide an additional measure of learning we also examined the latency to enter the food cup following CS presentations. The average latency to enter the food cup following the onset of the CS+ decreased across training blocks and this decrease did not differ between groups, demonstrating that all groups were similarly motivated to respond to reward-predictive cues (two-way repeated-measures ANOVA, main effect of training block: *F*_(5,225)_ = 16.95, *p* < 0.0001; n.s. main effect of group: *F*_(3,45)_ = 1.239, *p* = 0.307; n.s. group × training block interaction: *F*_(15,225)_ = 0.3964, *p* = 0.9791; Fig. 4*E*). In contrast, the average latency to enter the food cup following the onset of the CS– increased across training blocks, and did not differ between groups (two-way repeated-measures ANOVA, main effect of training block: *F*_(5,225)_ = 12.38, *p* < 0.0001; n.s. main effect of group: *F*_(3,45)_ = 0.6639, *p* = 0.578; n.s. group × training block interaction: *F*_(15,225)_ = 0.4812, *p* = 0.9485; Fig. 4*F*). Together, the results from these behavioral studies show that introduction of Cre into either D1- or A2a neurons does not disrupt normal acquisition or expression of instrumental and Pavlovian discriminations.

**Figure 5.**
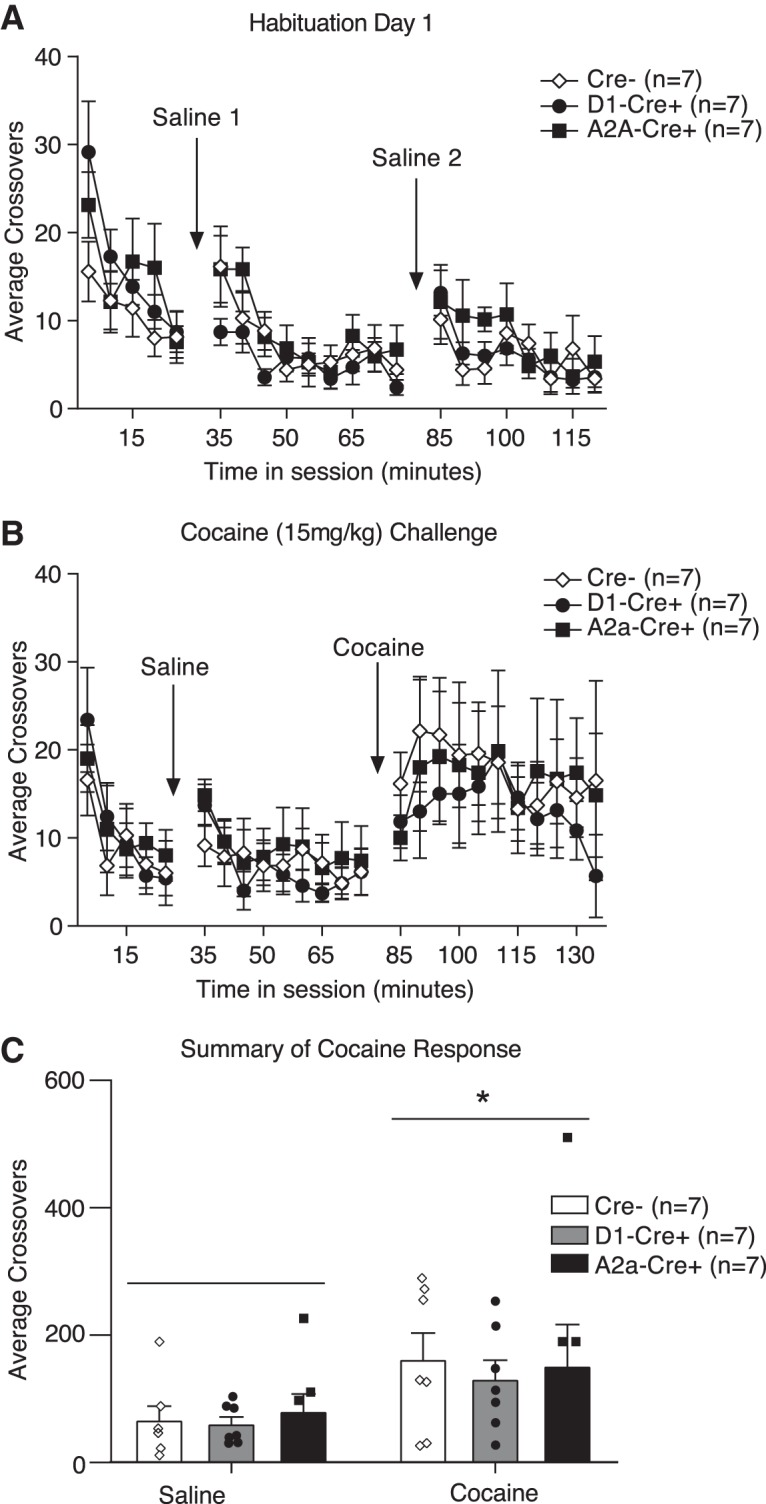
Basal and cocaine induced locomotor activity is similar between transgenic lines and Cre– littermate controls. ***A***, Locomotor activity decreases similarly in all groups across habituation and repeated saline injection. ***B***, Acute cocaine injection results in an increase in locomotor activity that is similar across groups. ***C***, Summary of locomotor activity in response to saline versus cocaine. Cocaine significantly increases locomotor activity compared to saline, and the magnitude of this response is similar across groups; *p < 0.005 locomotor activity in response to cocaine versus saline. All data represented as mean ± SEM.

Locomotor habituation and cocaine-induced locomotor activity were used to assess general striatal function in both lines (Oginsky et al., 2016). Cre*+* rats and their Cre– littermates were placed in standard locomotor chambers equipped with photocell beams around the perimeter. After a 30-min habituation period, they were given two intraperitoneal injections of saline (1 ml/kg). Both lines showed typical habituation to the locomotor chambers, and short-lived responses to saline injection that decreased with repeated injection (two-way repeated-measures ANOVA, main effect of time: *F*_(21,378)_ = 10.42, *p* < 0.0001; Fig. 5*A*). Locomotor activity was similar across D1-Cre and A2a-Cre lines and between Cre+ and Cre– rats (two-way repeated-measures ANOVA, n.s. effect of genotype: *F*_(2,18)_ = 0.3965, *p* = 0.6784; Fig. 5*A*). The next day, rats were again placed in locomotor chambers and given an injection of saline followed 40 min later by cocaine (15 mg/kg, i.p.). As expected, cocaine significantly increased locomotor activity, and the magnitude and time course of this response was similar between Cre*+* and Cre– rats, as well as across transgenic lines [two-way repeated-measures ANOVA, main effect of time: *F*_(23,414)_ = 6.901, *p* < 0.0001; n.s. effect of genotype: *F*_(2,18)_ = 0.09284, *p* = 0.9118 (Fig. 5*B*); two-way repeated-measures ANOVA, main effect of injection: *F*_(1,18)_ = 10.51, *p* = 0.0045; n.s. effect of genotype: *F*_(2,18)_ = 0.1284, *p* = 0.8803; n.s. injection by genotype interaction: *F*_(2,18)_ = 0.1122, *p* = 0.8945 (Fig. 5*C*)]. Thus, all genotypes showed a significant increase in locomotor activity following cocaine versus saline injection, and this effect did not differ between genotypes. These data suggest that there is no overt striatal dysfunction due to Cre expression, and that behavioral responses to elevations in dopamine are similar across D1-Cre and A2a-Cre lines.

## Discussion

We have demonstrated successfully targeted, functional knock-in of Cre recombinase at the *Drd1a* and *Adora2a* loci, without off-target insertions as assessed by multiple methods including whole-genome sequencing. Comparable behavioral performance across lines and between Cre*+* and Cre– littermates in several basic behavioral procedures provides further confidence that there are no unexpected deleterious effects of genetic manipulation or co-production of Cre recombinase with endogenous receptors. Within striatum we showed that Cre expression was consistent and selective to the correct populations of direct pathway D1+ and indirect pathway A2a+ cells, respectively. Thus, these D1-Cre and A2a-Cre transgenic rats enable selective monitoring or manipulation of dMSNs and iMSNs with high specificity. Although we fully expect Cre to be correctly targeted in other brain regions too, further characterization will be required to confirm this.

D1-Cre and A2a-Cre transgenic rats offer clear advantages over currently available transgenic models. First, the greater capacity of rats to learn complex behaviors make them stronger candidates for a wider range of tasks compared to mice. Second, the increased carrying capacity afforded by rats facilitates the chronic implantation of larger devices (i.e., high channel-count headstages, graded-refractive-index lenses). Thirdly, knock-ins can be used with higher confidence that the genetic modification was selective and specific to the target, compared to BAC lines.

A long-standing question in basal ganglia research has been the degree to which the striatal MSNs population can be fully divided into distinct D1+ and D2+/A2a+ subpopulations. Based on BAC transgenic mice overlap has been reported to range from 4% to 5% in DS and nucleus accumbens core, and up to 17% in shell (Bertran-Gonzalez et al., 2008; Wei et al., 2018). Our quantification of *Drd1* and *Adora2a* mRNA expression found overlap to be consistently very low in all striatal subregions examined, including shell, providing additional evidence for a fundamentally segregated striatal architecture.

Since Cre expression was highly specific to the intended striatal pathways, these rats are powerful tools for pathway-specific neuron identification and manipulations. One caveat is that a subset of fast-spiking, parvalbumin-positive (PV+) interneurons also express D1 receptors (Bracci et al., 2002), and may thus also express Cre in D1-Cre rats. However, PV+ are only ∼0.7% of striatal neurons (Luk and Sadikot, 2001) and at least in electrophysiological studies can be readily differentiated from MSNs (Kawaguchi, 1993; Koós and Tepper, 1999; Berke, 2008).

We chose to examine behavior during simple instrumental and Pavlovian tasks as well as cocaine-induced locomotor activity, as these behaviors rely heavily on striatal function. Although behavioral differences might emerge under other, more complex task conditions, the lack of any overt differences between the D1-Cre and A2a-Cre transgenic lines, or between Cre– and Cre*+* littermates, strongly suggest that these rats are well-suited for behavioral and systems neuroscience studies. Beyond striatum, A2a receptors are found in the cortex, globus pallidus, hippocampus, thalamus, cerebellum (Rosin et al., 1998), and throughout the cardiovascular system. Similarly, D1 receptors are located in prefrontal cortex, hippocampus, thalamus, and hypothalamus (Fremeau et al., 1991). In coordination with a rapidly expanding set of optical and genetic tools, these rats increase our ability to address fundamental questions about brain circuitry and mechanisms underlying neurologic and psychiatric disorders.

**Table 1.** G0 founder screening primers

Primer target	Line	Forward sequence	Reverse sequence
*iCre* internal	A2a-Cre; D1-Cre	AATGTGAACATTGTGATGAACTACA	CAGAATAGAATGACACCTACTCAGACA
insertion spanning 5' junction	A2a-Cre	AGGCAACTTTCTAGTTGACAAATCAAG	CAGCAGGCTGAAGTTAGTAGCTC
insertion spanning 3' junction	A2a-Cre	CATTGTCTGAGTAGGTGTCATTCTATTCT	GAATCACAGCCCAAGAGATACTACACT
insertion spanning 5' junction	D1-Cre	AAAAGTGACTAGAATTGACCTGGAAGAG	AGCAGGTTGGAGACTTTCCTCTTCTTCTT
insertion spanning 3' junction	D1-Cre	CATTGTCTGAGTAGGTGTCATTCTATTCT	GGAAAAGGAAAGAGAAGCAGAATAAT

**Table 2.** Genotyping primers

Primer target	Line	Forward sequence	Reverse sequence	Reporter 1
Insertion spanning	A2a-Cre	CGTCTCCAGCCTGCTTCAG	TCCTCATGGTCTTCAGAGTTTGC	CCGGAAGCGGAGCTAC
Insertion spanning	D1-Cre	GTGAGGCTGCTCGAGGAT	CTGGCAACTAGAAGGCACAGT	CCTGGACAGCACCTGAC

## Synthesis

Reviewing Editor: Yavin Shaham, NIDA-IRP/NIH Decisions are customarily a result of the Reviewing Editor and the peer reviewers coming together and discussing their recommendations until a consensus is reached. When revisions are invited, a fact-based synthesis statement explaining their decision and outlining what is needed to prepare a revision will be listed below. The following reviewer(s) agreed to reveal their identity: Bruce Hope, Brandon Harvey.

The reviewers found significant merit in the study but also have raised several concerns that should be addressed in the revision. It is particularly important to address with either text changes or additional validation data the comments of reviewer 2. I enclose below the individual comments of the reviewers and below each of them my recommendation of how to address these comments.

REVIEWER 1

The authors describe new D1-Cre and A2a-Cre transgenic rats that were made using targeted insertions at the 3'-end of the endogenous genes. They provide a very thorough confirmation of correct insertion into the genome. The FISH confirmed localization to the correct D1 and A2a-expressing neurons. Cre was expressed at both the mRNA and protein levels to permit the opsin activation and function. So far, the existing data indicates no obvious interference with behavior. Overall these rats will be very important for assessing neural mechanisms of learning and behavior. There are many differences in the neural mechanisms between mice and rats including that of the D1 and D2/A2a localization and function, and rats demonstrate higher levels of cognitive abilities. I have only minor comments.

1-Please explain why you did not assess cortical or other brain areas. Are you going to assess these in another study?

Editor: please address this issue with text changes in the Discussion

2-The abstract, introduction and methods gave me the impression that you were only going to assess the dorsal striatum. You should mention that you are examining the accumbens in these sections.

Editor: please address with text changes in the Introduction

3-The citations need to be better sourced. Find the original citations for a given claim. For example, in the second sentence of the introduction, the authors cite Berke 2018 for the role of striatum in movement, motivation and reinforcement-driven learning. The correct citations come from 1970s and 80s, and if they want to cite a recent overview they should cite the people who actually created a significant amount of the underlying work. To this end, Gerfen and Surmeier are the originals, so this citation was fine. There are a number of these citations in the manuscript where the authors just chose whatever recent paper they found without going to the original sources or researchers that did the work.

Editor: please include relevant historical citations in the Introduction and Discussion

REVIEWER 2

1. What generation of rats was blood sample taken from for genome sequencing? Not listed in Methods under “Genome sequencing was performed using” section.

Editor: please add the information to the Methods

2. It appears animals were crossed with WT LE and heterozygous animals used. If so, the statement in results that a 1:1 stoichiometry of “gene” mRNA to Cre mRNA would be produced. It should be 2:1 assuming that both alleles are transcribed equally. This does not appear to be what was obtained. Considering doing qPCR for Cre and corresponding target gene mRNA .

Editor: Please address this comment with text changes and if possible additional data. However, additional data are not critical to a successful revision

3. Use of a 2A is not 100% efficient in cleaving the translational fusion protein. A western blot to Cre should be performed. More importantly, a western blot probing D1 and A2a should be performed to show that protein levels are similar to WT animals.

Editor: Pease consider adding empirical data to address this important validation issue

4. Is there evidence that the 2a residues left on the C-terminus of either D1 or A2a alter their function? This should be determined to rule out creating dominant negatives or altered D1 or A2a for all subsequent studies using these animals. At minimal, this should be discussed in context of tagged D1 and A2a receptors.

Editor: We recommend either addressing this validation issue in the Discussion or add new data to empirically address this comment

5. A Cre-dependent virus is injected into 3 areas where Cre is expected, what about other regions? What does Cre activity and/or expression look like in other regions where Cre is expected or not expected to be expressed based on what is known about endogenous D1 or A2a expression. For example, in Discussion many other regions expressing these genes are listed, what does Cre expression look like in these regions?

Editor: Additional data from other brain areas is not required for the revision. Please address this issue in the Discussion

6. Consider putting DNA sequences into a Table.

Editor: please add this information
